# Uniportal versus multiportal video-assisted thoracoscopic surgery for spontaneous pneumothorax

**DOI:** 10.1186/s13019-024-02931-4

**Published:** 2024-06-26

**Authors:** Nicky Janssen, Aimée J.P.M. Franssen, Arlette A. Ramos González, Iris E.W.G. Laven, Yanina J.L. Jansen, Jean H.T. Daemen, Pieter W.J. Lozekoot, Karel W.E. Hulsewé, Yvonne L.J. Vissers, Erik R. de Loos

**Affiliations:** Department of Surgery, Division of General Thoracic Surgery, Zuyderland Medical Center, Henri Dunantstraat 5, Heerlen, 6419PC The Netherlands

**Keywords:** Spontaneous pneumothorax, Video-assisted thoracoscopic surgery, Bullectomy, Pleurectomy

## Abstract

**Background:**

Multiportal video-assisted thoracic surgery (mVATS) is the standard approach for the surgical treatment of spontaneous pneumothorax. However, uniportal VATS (uVATS) has emerged as an alternative aiming to minimize surgical morbidity. This study aims to strengthen the evidence on the safety and efficiency of uVATS compared to mVATS.

**Methods:**

From January 2004 to December 2020, records of patients who had undergone surgical treatment for primary or secondary spontaneous pneumothorax were evaluated for eligibility. Patients who had undergone pleurectomy combined with bullectomy or apical wedge resection via uVATS or mVATS were included. Surgical characteristics and postoperative data were compared between patients who had undergone surgery via uVATS or mVATS. Univariable and multivariable analyses were performed to determine whether the surgical approach was associated with any complication (primary outcome), major complications (i.e., Clavien-Dindo *≥* 3), recurrence, prolonged hospitalization or prolonged chest drainage duration (secondary outcomes).

**Results:**

A total of 212 patients were enrolled. Patients treated via uVATS (*n* = 71) and mVATS (*n* = 141) were significantly different in pneumothorax type (secondary spontaneous; uVATS: 54 [76%], mVATS: 79 [56%]; *p* = 0.004). No significant differences were observed in (major) complications and recurrence rates between both groups. Multivariable analyses revealed that the surgical approach was no significant predictor for the primary or secondary outcomes.

**Conclusions:**

This study indicates that uVATS is non-inferior to mVATS in the surgical treatment of spontaneous pneumothorax regarding safety and efficiency, and thus the uVATS approach has the potential for further improvements in the perioperative surgical care for spontaneous pneumothorax.

## Introduction

Spontaneous pneumothorax can be classified as primary spontaneous pneumothorax (PSP) or secondary spontaneous pneumothorax (SSP) and treated with surgical and non-operative options, depending on the subtype, recurrence status, or patient characteristics. Surgical intervention entails some type of pleural intervention (i.e., talk pleurodesis, pleurectomy or pleural abrasion) combined with a bullectomy (or apical wedge resection). While the optimal surgical treatment for PSP is still a matter of debate [1], non-surgical treatments (e.g., aspiration, chest tube) lead to considerably high recurrence rates, ranging from 21 to 30% within the first year following the first episode [2–5]. Recurrence rates are even higher after a second episode, approximately 60% [5]. Therefore, surgical treatment for recurrent PSP and first-episode PSP with prolonged air leak is advised [6]. For patients with SSP, surgical intervention is advised upon their first episode as their lung function is already compromised.

Over the past two decades, video-assisted thoracic surgery (VATS) has revolutionized thoracic surgery. VATS is a minimally invasive technique offering numerous advantages in terms of postoperative morbidity compared to thoracotomy [7–9] and is therefore nowadays considered as gold standard [10, 11]. Initially in our hospital, VATS was performed via a multiport approach (mVATS). As of 2016, VATS via a single port (uniportal VATS [uVATS]) was implemented, aiming to minimize surgical morbidity while maximizing efficacy [12–14].

To date, all but two studies solely focus on the PSP subgroup in comparing uVATS to mVATS for the treatment of spontaneous pneumothoraces. These studies were, however, limited by their small sample size of only 35 and 24 patients [15, 16]. In this retrospective cohort study, we present our 5-year experience with uVATS for spontaneous pneumothorax and compare these perioperative outcomes with patients who had undergone surgery for spontaneous pneumothorax via the mVATS approach, aiming to strengthen the evidence on the safety in terms of (major) complications and efficiency (recurrence, chest tube drainage duration and hospitalization) of uVATS.

## Methods

### Study design, setting and patient selection

A single center retrospective cohort study was conducted at Zuyderland Medical Center, Heerlen, the Netherlands. Medical records of all consecutive patients who had undergone VATS for spontaneous pneumothorax between January 2004 and December 2020 were retrospectively reviewed for eligibility. Patients were included if they had undergone a pleurectomy combined with bullectomy or apical wedge resection. As the uVATS approach was implemented in April 2016, these patients were compared to patients who had undergone mVATS between January 2004 and April 2016. Patients eligible for such a procedure encompassed those with first episode PSP with prolonged air-leakage after chest tube placement, recurrent PSP with or without the existence of bulla or blebs on computed tomography (CT), or SSP. SSP was defined as a pneumothorax with chronic lung disease or systemic disease with lung involvement as the underlying cause (i.e., emphysema, chronic obstructive pulmonary disease (COPD), interstitial lung disease (ILD), tuberculosis, and lung cancer) based on preoperative diagnostics (including imaging and the patient’s disease history) or the pathology report. In patients aged 50 years or older with a smoking history, pneumothorax was also deemed secondary, in accordance with the British Thoracic Society pleural disease guideline [6]. All other patients with a spontaneous pneumothorax and no known underlying cause were classified as PSP. Patients were excluded in case of a primary open approach, other or concurrent treatments (i.e., lobectomy, pleurodesis, fibrin glue), or missing surgical procedure or follow-up data. If a patient had an ipsilateral recurrent pneumothorax within 30 days and received surgical treatment for both episodes, the patient was only included once (i.e., inclusion at the first episode). The surgical procedures were performed by qualified and experienced thoracic surgeons.

The study protocol was approved by the local medical ethics and research committee of Zuyderland Medical Center and Zuyd University of Applied Sciences (METC Z; registration number: METCZ20200097; date of approval: June 12, 2020), and the need for individual informed consent was waived. The study was conducted in accordance with the Declaration of Helsinki (as revised in 2013). This report was written in accordance with the Strengthening the Reporting of Observational studies in Epidemiology (STROBE) guidelines [17].

### Surgical approach and technique

For the uVATS approach, a single 3–4 cm incision in the fourth or fifth intercostal space, between the mid-axillary and anterior axillary lines is made. For the mVATS approach, the port placements are as follows:

- 12 mm port in the ninth intercostal space for video guidance.
- 12 mm port in the seventh to eighth intercostal space at the anterior axillary line.
- A trocar in the sixth intercostal space at the level of the tip of the scapula posteriorly.

**Fig. 1:**
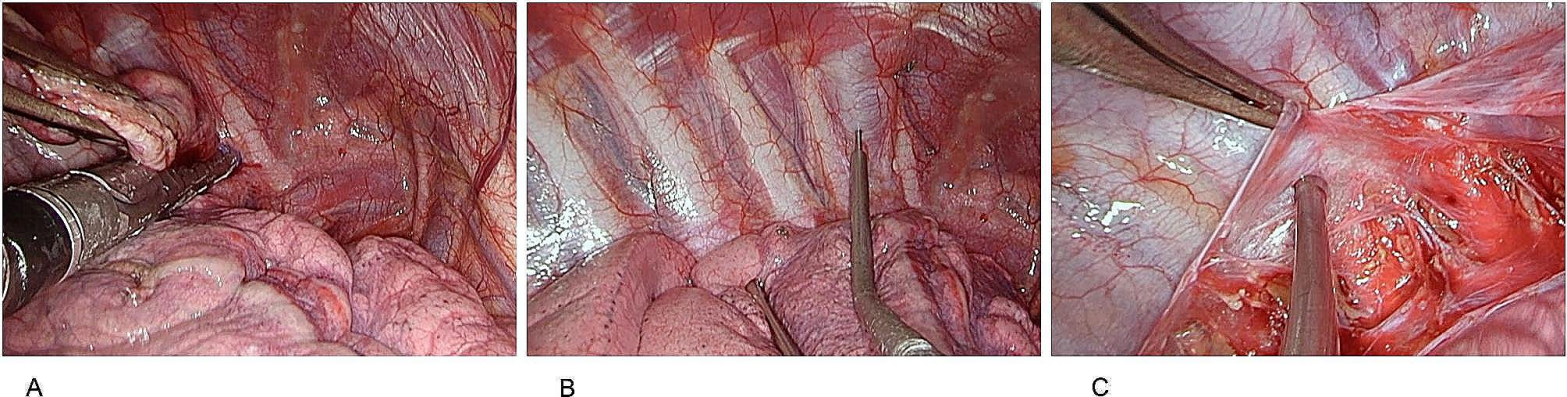
Surgical technique. (**A**) Excision of bullae using a linear endostapler. (**B**) A saline solution is injected under pressure using a long thoracoscopic needle causing separation of the parietal pleura from the chest wall. (**C**) The parietal pleura is removed using a long thoracoscopic forceps. The blunt tip of a suction probe is used to assist in the dissection

The latter is excluded with the two-port approach. Bullae were excised using a linear endostapler with tissue-specific cartridges (Echelon™, Ethicon Endo-Surgery, Johnson and Johnson, Amersfoort, the Netherlands)(Fig. 1A). If no blebs or bullae were visible at CT imaging and during visual inspection of the lung, an apical wedge resection was performed. Since blebs and bullae most commonly occur at the apex of the lung, this approach aims to reduce the risk of recurrence by addressing potential sites of occult pathology and stimulating adhesion of the apex of the upper lobe to the apical chest wall. In addition, a pleurectomy was performed with the use of hydrodissection of the parietal pleura (Fig. 1B-C). The parietal pleura was resected from the second intercostal space to the level of the diaphragm, avoiding dissection near the brachiocephalic trunk and subclavian neurovascular structures. Mediastinal and diaphragmatic pleura were not resected. After haemostasis and placement of a Ch28 chest tube, the wounds were closed in layers. In some cases, an extra chest tube was placed at discretion of the surgeon. Water-seal was applied for chest drainage. A plain chest radiograph was routinely acquired at the recovery unit. Postoperative physical therapy including breathing exercises was started on the first postoperative day.

### Variables and data collection

Electronic patient files were retrospectively reviewed for patient characteristics including age, sex, body mass index (BMI), smoking status, American Society of Anesthesiologists (ASA) classification, affected side, type of spontaneous pneumothorax (i.e., PSP or SSP) and surgical indication (i.e., first episode PSP with prolonged air leak, recurrent PSP or SSP). Obtained procedural characteristics included type of approach (i.e., uVATS/mVATS), type of surgery (i.e., pleurectomy combined with bullectomy or apical wedge resection), year of surgery, operation time, and conversion (i.e., to thoracotomy or from uVATS to mVATS). Furthermore, postoperative outcomes encompassed postoperative length of hospital stay (days), postoperative chest drainage duration (days), complications, intensive care unit (ICU) admission due to complication or conversion, readmission within 30 days, prolonged air leak requiring reintervention (i.e., chest drain, chemical pleurodesis, additional bullectomy) and recurrent ipsilateral pneumothorax requiring intervention. If multiple chest tubes were placed, the drainage duration was determined by the removal of the last chest tube. Prolonged chest drainage duration was defined as a chest drainage period longer than 5 days based on the median chest drainage period after conventional mVATS pleurectomy with bullectomy at our institution. Prolonged hospitalization was defined as a postoperative hospitalization longer than 6 days, based on the aforementioned chest drainage duration as patients are normally discharged 1 day after drain removal. Complications were recorded and classified according to the Clavien-Dindo classification into major (i.e., grade 3 or higher) or minor (i.e., lower than grade 3) [18].

The primary endpoint was defined as any complication within 30 days after surgery. Secondary outcomes included major complications as defined by Clavien-Dindo ≥ 3, recurrence, postoperative length of hospital stay, and chest drainage duration. The follow-up period is expressed in days and was recorded until the patient’s last outpatient clinic visit to the lung surgery or pulmonology department at the time of data entry.

### Statistical analysis

Data analysis was performed using SPSS (IBM Corp. Released 2019. IBM SPSS Statistics for Windows, Version 26.0. Armonk, NY: IBM Corp). Normal distribution of continuous variables was assessed by the Kolmogorov-Smirnov test and visual assessment of normal-probability plots. In the absence of normality, data was depicted as median and interquartile range (IQR) and differences between groups were evaluated by the Mann-Whitney U test. Normal distributed data was reported as mean and standard deviation (SD). Categorical variables were depicted as frequency and percentage and compared by the Chi-square or Fisher’s exact test where appropriate. Values of *p* < 0.05 were considered statistically significant and missing data was reported as such.

Univariable and multivariable regression analyses were performed to determine the impact of the surgical approach (mVATS vs. uVATS) on each primary and secondary endpoint, including any complication, major complications, prolonged hospitalization and chest drainage duration, and recurrence. Unadjusted univariable analyses were performed for surgical approach and the following baseline covariates: age, sex, BMI, history of smoking, ASA classification, type of pneumothorax and year of surgery. To assess the impact of the surgical approach on all endpoints after adjustment for baseline covariates, multivariable logistic regression analyses were performed. In case of missing data for any of the independent variables, the patient was excluded from the multivariable analysis. Backward stepwise elimination was applied to produce the most accurate model and predictors were removed if the p-value was greater than 0.1. In the final model, independent variables with a p-value less than 0.05 were considered as a significant predictor for the endpoint. Odds ratios (OR) were calculated with confidence intervals (95% CI).

## Results

### Participants

**Fig. 2:**
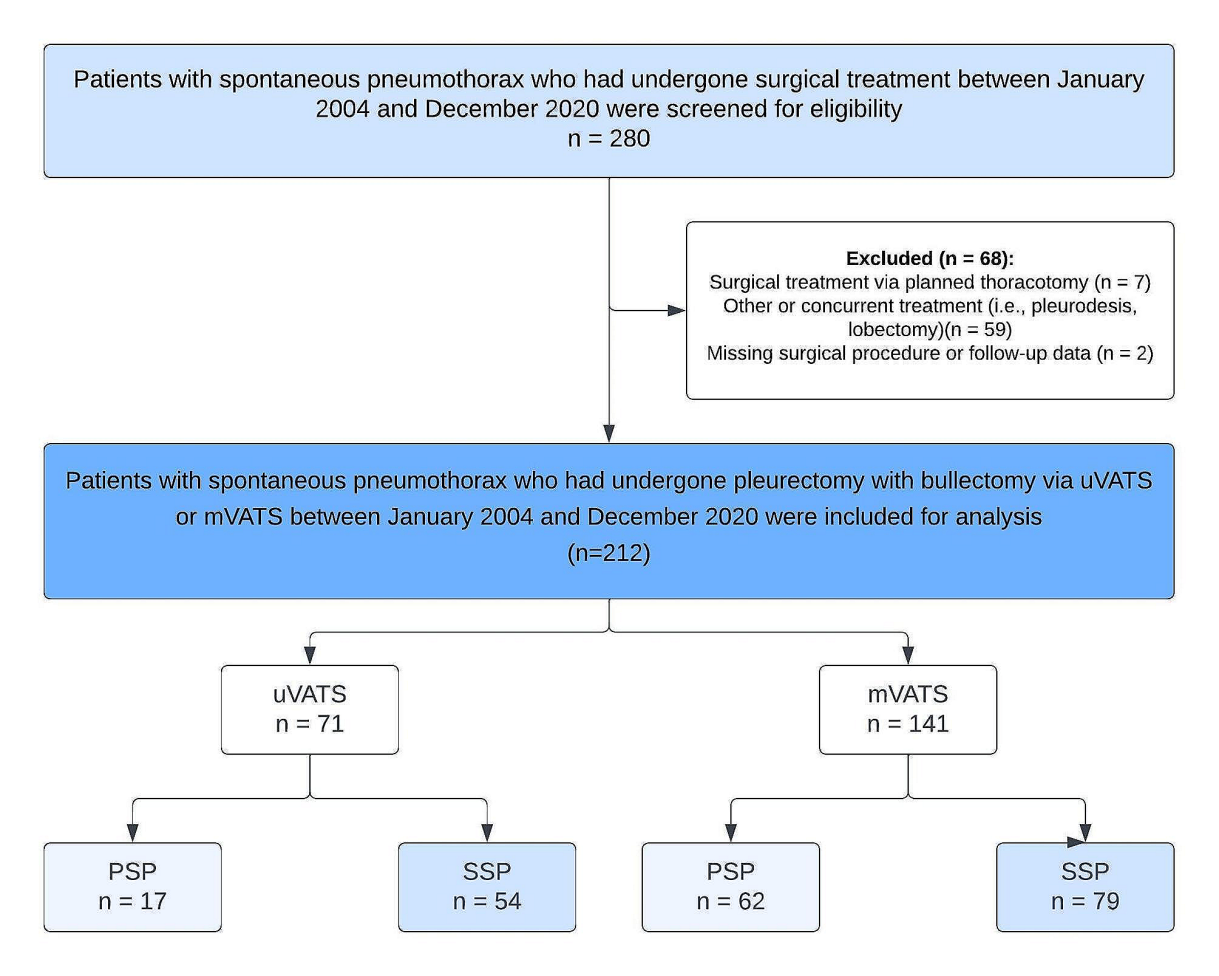
STROBE flowchart of patient selection. *mVATS = Multiportal video-assisted thoracoscopic surgery; PSP = primary spontaneous pneumothorax; SSP = secondary spontaneous pneumothorax; uVATS = uniportal video-assisted thoracoscopic surgery*

A total of 280 potentially eligible patients were identified (Fig. 2). Sixty-eight patients were excluded based on the previously mentioned exclusion criteria (i.e., planned thoracotomy, *n* = 7; other or concurrent treatment, *n* = 59; missing surgical procedure data, *n* = 2). The remaining 212 patients were included for analysis, of whom 71 (33%) in the uVATS group and 141 (67%) in the mVATS group. Baseline characteristics of both groups are displayed in Table 1. The uVATS group consisted of significantly more patients with the SSP subtype than the mVATS group (uVATS 54 [76%], mVATS 79 [56%]; *p* = 0.004) and correspondingly the surgical indication. No significant differences were found in other baseline characteristics. Patients in both groups were predominantly male, 47 (66%) in the uVATS group and 105 (75%) in the mVATS group, with a median age of 36.4 (IQR 22.3–53.5) in the uVATS group and 39.0 (IQR 26.3–53.9) in the mVATS group. Median follow-up period in the mVATS group was 825 (IQR 203–3126) days and 247 (IQR 97-1456) days in the uVATS group.

**Table 1:** Baseline patient characteristics

Characteristics	Surgical Technique				
	uVATS (*n* = 71)	Missing data, *n* (%)	mVATS (*n* = 141)	Missing data, *n* (%)	*p*-value
**Age**, y, median (IQR)	36.4 (22–54)		39.0 (26–54)		0.33
**Sex**, male	47 (66)		105 (75)		0.21
**BMI**, median (IQR)	20.8 (19-23)	1 (1)	21.6 (19-25)	1 (1)	0.18
**Smoking**				13 (9)	0.33
Yes	44 (62)		66 (52)		
No	15 (21)		31 (24)		
Stop	12 (17)		31 (24.2)		
**ASA classification**				4 (3)	0.403
1	24 (34)		58 (42)		
2	31 (44)		54 (39)		
3	16 (23)		23 (17)		
4	0 (0)		2 (2)		
**Type of pneumothorax**					**0.004**
PSP	17 (24)		62 (44)		
SSP	54 (76)		79 (56)		
**Surgical indication**					**0.02**
First episode PSP with prolonged air leak	7 (10)		22 (16)		
Recurrent PSP	10 (14)		40 (28)		
SSP	54 (76)		79 (56)		
**Affected side**					0.74
Right	45 (63)		86 (61)		
Left	26 (37)		55 (39)		

Values are n (%) unless otherwise indicated

ASA: American Society of Anesthesiologists physical status; BMI: body mass index; IQR: interquartile range; mVATS: multiportal video-assisted thoracoscopic surgery; PSP: primary spontaneous pneumothorax; SSP: secondary spontaneous pneumothorax; uVATS: uniportal video-assisted thoracoscopic surgery

### Surgical characteristics

Surgical characteristics are listed in Table 2. The majority of the patients had undergone a pleurectomy combined with a bullectomy (uVATS: 63 [89%], and mVATS: 122 [87%]; *p* = 0.65). No statistical difference was observed for the length of operation between uVATS and mVATS, with median surgery duration of 61 min (IQR 48.0-74.5) and 60 min (IQR 50.0–70.0), respectively.

**Table 2:** Procedural characteristics

Characteristics	Surgical Technique				
	uVATS (*n* = 71)	Missing data, *n* (%)	mVATS (*n* = 141)	Missing data, *n* (%)	*p*-value
**Type of surgery**					0.65
Bullectomy and pleurectomy	63 (89)		122 (87)		
Apical wedge resection and pleurectomy	8 (11)		19 (14)		
**Operation time**, min, median (IQR)	61 (48–75)	2 (3)	60 (50–70)	22 (16)	0.37
**Conversion**					
to thoracotomy	0 (0)		4 (3)		0.30
to mVATS	1 (1)		NA		

Values are n (%) unless otherwise indicated

IQR: interquartile range; mVATS: multiportal video-assisted thoracoscopic surgery; NA: not applicable; post-op: postoperative; uVATS: uniportal video-assisted thoracoscopic surgery

In the mVATS group, four (3%) procedures were converted to thoracotomy to enhance surgical exposure and three of these patients were transferred to the ICU for postoperative monitoring. One out of the four conversions to thoracotomy was in a patient with recurrent PSP, with the remaining conversions occurring in patients with SSP and one case of PSP with a prolonged air leak. In the uVATS group, only one (3%) conversion to mVATS was required to enhance surgical exposure in a patient with SSP, and no conversions to thoracotomy were performed. However, the difference in conversion rate was not statistically significant (*p* = 0.14).

### Postoperative results

Postoperative results are listed in Table 3. A significant difference in postoperative chest drainage duration (uVATS: 4 days [IQR 3.0–6.0], mVATS: 5 days [IQR 4.0–7.0]; *p* = 0.01]) and postoperative length of hospital stay (uVATS: 5 days [IQR 4.0–7.0], mVATS: 6 days [IQR 4.0-8.5]; *p* = 0.005) was observed between both groups, with a median decrease of 1 day in the uVATS group on both variables.

**Table 3:** Postoperative outcomes

Variables		Surgical technique				
	uVATS (*n* = 71)		Missing data, *n* (%)	mVATS (*n* = 141)	Missing data, *n* (%)	*p*-value
**Postoperative chest drainage duration**, d, median (IQR; range)	4 (3-6)			5 (3-7)		**0.01**
**Postoperative length of hospital stay**, d, median (IQR; range)	5 (4-7)			6 (4-9)		**0.005**
**Major complications (≥** **3)**	8 (11)			17 (12)		> 0.99
Persistent air leak requiring intervention	4 (6)			10 (7)		
Hemothorax requiring intervention	1 (1)			4 (3)		
Empyema requiring re-VATS	1 (1)			2 (1)		
Respiratory insufficiency requiring intubation	0 (0)			1 (1)		
Atelectasis requiring bronchoscopy	2 (3)			0 (0)		
**Minor complications (< 3)**	0 (0)			4 (3)		0.30
Pneumonia requiring antibiotics	0 (0)			3 (2)		
Wound infection requiring antibiotics	0 (0)			1 (1)		
**ICU admission**						0.37
Due to conversion	0 (0)			3 (2)		
Due to complication	1 (1)			4 (3)		
**Recurrence**	4 (6)			9 (6)		> 0.99
**Re-admission within 30 days**	3 (4)			3 (2)		0.69

Values are n (%) unless otherwise indicated

ICU: intensive care unit; IQR: interquartile range; re-VATS: redo video-assisted thoracoscopic surgery

Postoperative (major) complication rates were comparable for both groups (any complication, *p* = 0.54; major complications, *p* > 0.99; Table 3). In the uVATS group seven (10%) patients had a total of eight complications, while in the mVATS group, 21 complications were reported in 21 (14%) patients. Seventeen (12%) patients in the mVATS group experienced a major complication that required an intervention, compared to eight (11%) patients in the uVATS group. A single patient, with several comorbidities including COPD gold 3, in the mVATS group had a complication with a Clavien-Dindo grade 4. This patient required reintubation because of postoperative respiratory insufficiency. All patients (uVATS: 1 [1%], mVATS: 4 [3%]; *p* = 0.67) with a haemothorax were admitted to the ICU for postoperative monitoring after surgical management of the bleeding. Other postoperative complications are further described in Table 3.

Number of recurrences was comparable between both groups (uVATS: 4 [6%], mVATS: 9 [6%]; *p* > 0.99). After discharge, three patients (4%) from the uVATS group were re-admitted within 30 days compared to three patients (2%) in the mVATS group, which was not significantly different between both groups (*p* = 0.69).

### Univariable and multivariable analysis

**Fig. 3:**
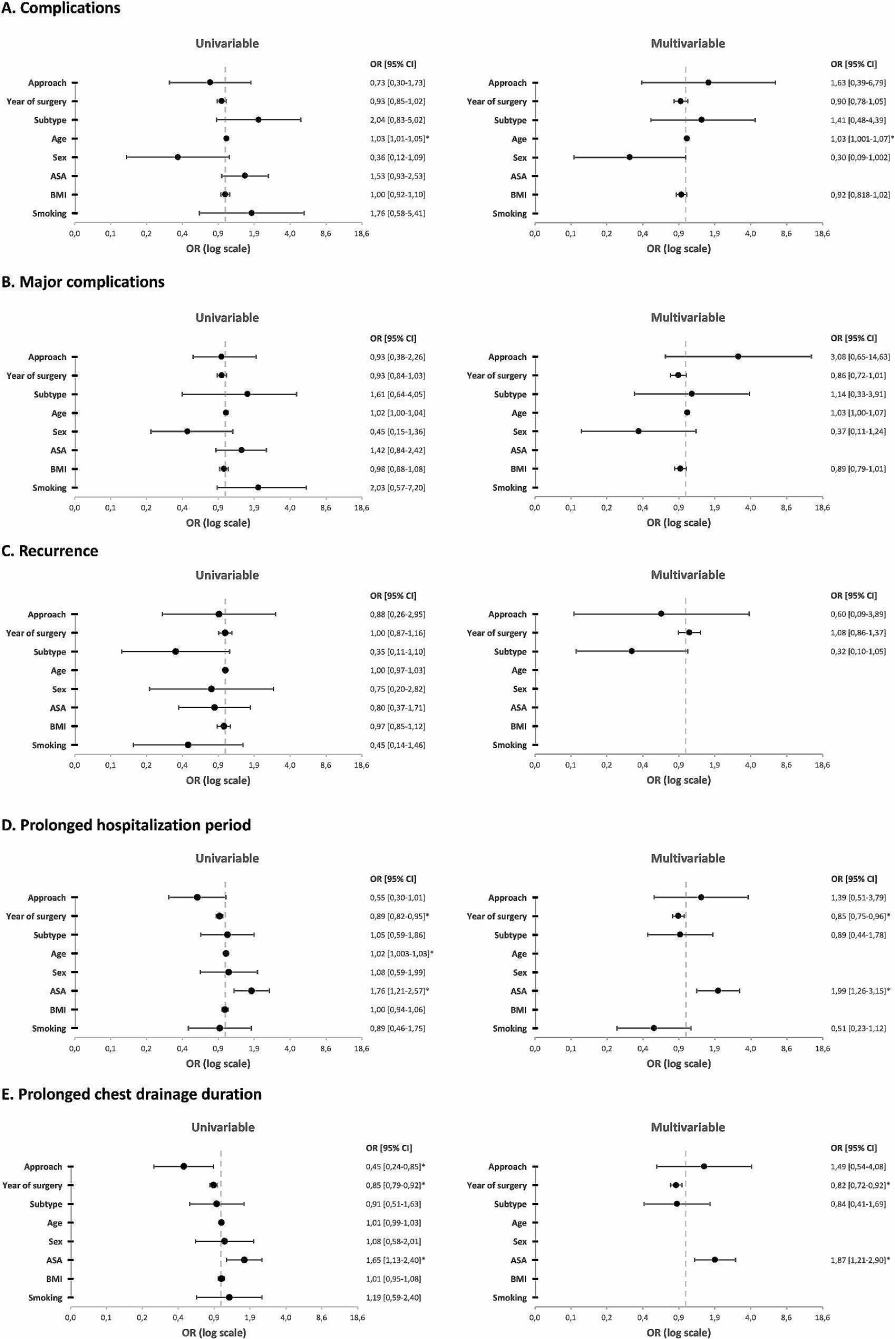
Uni- and multivariable analysis. * = p-value < 0.05; CI = confidence interval; OR = odds ratio

After excluding patients with missing data (*n* = 18), multivariable analysis identified age as a minor risk factor for postoperative complications (OR 1.03, 95% CI [1.001–1.07]) and major postoperative complications (OR 1.03, 95% CI [1.004–1.07]. No other risk factors for (major) postoperative complications or recurrence were found (Fig. 3A-C). However, prolonged hospitalization and prolonged chest drainage were both significantly influenced by the year of surgery indicating that more recent surgical procedures are linked to an improved outcome (OR 0.85 [95% CI, 0.75–0.96]; and OR 0.82 [95% CI, 0.72–0.92]) (Fig. 3D-E). Furthermore, ASA classification was significantly associated with a prolonged hospitalization and chest drainage duration (OR 1.99 [95% CI, 1.26–3.15); and OR 1.87 [95% CI, 1.21–2.90]). Overall, the surgical approach was no significant predictor for any type of complication or recurrence.

## Discussion

Surgical intervention remains the treatment of choice for recurrent or persistent PSP and SSP [19]. Recent developments in thoracoscopic surgery demonstrated a shift towards less invasive techniques, replacing the conventional mVATS approach with uVATS [20]. Our study showed no significant differences in postoperative complication or recurrence rates between patients who underwent surgical treatment for SSP and PSP via a uVATS or mVATS approach. In addition, the surgical approach was not significantly associated with prolonged hospitalization or chest drainage duration. Multivariable analyses did demonstrate age as risk factor for the development of (major) postoperative complications, as well as ASA classification for prolonged hospitalization and chest drainage duration.

It’s important to note that limited literature exists specifically focusing on secondary spontaneous pneumothorax (SSP), which was included in our study cohort. The most recent meta-analysis by Xu et al. (2017) on the treatment of the PSP subgroup suggests superiority of uVATS in postoperative morbidity and reveals comparable recurrence rates between the surgical approaches [21]. Standardized mean difference in length of hospital stay was − 0.39 days and − 0.68 days in chest drainage duration. Studies conducted after this meta-analysis, including a propensity matched study of prospectively collected data by Nachira et al. (2018), confirm these results [16, 22–27]. This is also in line with the meta-analysis by Abouarab et al. (2018), evaluating the effect of uVATS on perioperative outcomes in the broad field of thoracic surgery [28]. A recent prospective randomized trial by Kutluk et al. (2018) confirmed non-inferiority in postoperative morbidity and recurrence rates for PSP [26]. Results in our study support the suggestion that uVATS is a safe (i.e., in terms of (major) complications) and efficient (i.e., in terms of recurrence, chest tube drainage duration and hospitalization duration) surgical approach, not only for PSP, but for the entire group of spontaneous pneumothoraces.

Even though our study reported no differences in recurrence rates between both groups, we report upper limit recurrence rates for both surgical approaches when compared to the abovementioned studies (4–6%). This could be related to the fact that the majority of included patients were treated for SSP which is known to be associated with higher recurrence rates [29]. Furthermore, in our study, cannabis use was not assessed due to a lack of data, while cannabis use is a well-known factor negatively influencing recurrence rates [30]. Results regarding differences in length of hospital stay and drainage duration are comparable with outcomes of the previously mentioned meta-analysis.

A striking finding of our study was the significant association between the year of surgery and the risk of prolonged hospitalization and chest drainage. If the surgery was performed more recently, a diminished risk of prolonged chest drainage duration or hospitalization was observed. This may have been attributable to the implementation of enhanced recovery after surgery (ERAS) protocols including a more progressive approach to drain management [31]. A shorter chest drainage duration will lead to a better mobility which may contribute to a faster patient recovery. Also, hospital costs are decreased due to a decrease in postoperative length of hospital stay.

This study was limited due to its single-center retrospective design. However, missing data was randomly distributed throughout the study sample, and multivariate analyses were performed to mitigate any potential bias induced by differences in population size, population heterogeneity and variations in perioperative protocols over the relatively long enrolment period of 17 years. It is worth noting that this study did not adhere to the recommendation of a minimum of 10 events per variable in the multivariable analysis, which could have led to overfitting. Nevertheless, because complication and recurrence rates are low, conducting studies with larger sample sizes would not have been clinically feasible. Furthermore, though propensity score matching could have provided additional insights into balancing groups, performing this analysis was not feasible given the low outcome rates, as it would result in a considerable loss of sample size, thereby impeding statistical power and limiting the ability to detect significant differences between groups. The median follow-up period differed between the two groups; however, this discrepancy does not impact the primary outcome, which was assessed within the initial 30 days post-surgery, and most secondary outcomes as they were measured during hospitalization period. Furthermore, variables on known additional advantages (i.e., decreased postoperative pain and paraesthesia related to the ports, and higher patient satisfaction) of uVATS were not assessed in the present study as this data was not available in the medical patient records [15, 21, 22]. Also, this study did not specifically define or exclude the learning curve period for the uniportal VATS (uVATS) approach, which could have influenced the results of the uVATS group. However, it is worth noting that the transition period from multiportal VATS (mVATS) to uVATS was relatively brief, as the uVATS technique was concurrently introduced in other surgical procedures at our hospital. Additionally, complications and recurrences were evenly distributed throughout the cohort, and procedure time for uVATS was notably short and stabilized after only a few procedures, indicating a rapid acquisition of proficiency and suggesting a steep learning curve for the uVATS approach.

## Conclusions

The uVATS approach is equally safe in terms of (major) complications and effective (i.e., in terms of recurrences, prolonged hospitalization and chest drainage duration) in the surgical treatment of spontaneous pneumothorax compared to mVATS. While uVATS may not confer the significant advantage over mVATS that mVATS had over the conventional thoracotomy in the past, this study highlights the potential of uVATS for further improvements in perioperative care of pneumothorax patients.

## Data Availability

The data that support the findings of this study are available from the corresponding author upon reasonable request.

## Data availability

The data that support the findings of this study are available from the corresponding author upon reasonable request.

## Abbreviations

ASA: American Society of Anaesthesiologists physical status
BMI: Body mass index
COPD: Chronic Obstructive Pulmonary Disease
ICU: Intensive care unit
IQR: Interquartile range
mVATS: Multiportal video-assisted thoracoscopic surgery
STROBE: Strengthening the reporting of observational studies in epidemiology
VATS: Video-assisted thoracoscopic surgery
uVATS: Uniportal video-assisted thoracoscopic surgery
